# Chondroitinase gene therapy improves upper limb function following cervical contusion injury

**DOI:** 10.1016/j.expneurol.2015.05.022

**Published:** 2015-09

**Authors:** Nicholas D. James, Jessie Shea, Elizabeth M. Muir, Joost Verhaagen, Bernard L. Schneider, Elizabeth J. Bradbury

**Affiliations:** a King's College London, Regeneration Group, The Wolfson Centre for Age-Related Diseases, Guy's Campus, London Bridge, London SE1 1UL, UK; b Department of Physiology, Development and Neuroscience, University of Cambridge, Cambridge CB2 3EG, UK; c Laboratory for Neuroregeneration, Netherlands Institute for Neuroscience, 1105BA Amsterdam, The Netherlands; d Brain Mind Institute, Ecole Polytechnique Fédérale de Lausanne, 1015 Lausanne, Switzerland

**Keywords:** Spinal cord injury, Cervical contusion, Gene therapy, Chondroitinase ABC

## Abstract

Chondroitin sulphate proteoglycans (CSPGs) are known to be important contributors to the intensely inhibitory environment that prevents tissue repair and regeneration following spinal cord injury. The bacterial enzyme chondroitinase ABC (ChABC) degrades these inhibitory molecules and has repeatedly been shown to promote functional recovery in a number of spinal cord injury models. However, when used to treat more traumatic and clinically relevant spinal contusion injuries, findings with the ChABC enzyme have been inconsistent. We recently demonstrated that delivery of mammalian-compatible ChABC via gene therapy led to sustained and widespread digestion of CSPGs, resulting in significant functional repair of a moderate thoracic contusion injury in adult rats. Here we demonstrate that chondroitinase gene therapy significantly enhances upper limb function following cervical contusion injury, with improved forelimb ladder performance and grip strength as well as increased spinal conduction through the injury site and reduced lesion pathology. This is an important addition to our previous findings as improving upper limb function is a top priority for spinal injured patients. Additionally great importance is placed on replication in the spinal cord injury field. That chondroitinase gene therapy has now been shown to be efficacious in contusion models at either thoracic or cervical level is an important step in the further development of this promising therapeutic strategy towards the clinic.

## 1 Introduction

Spinal cord injury (SCI) is a devastating condition, leading to varying degrees of motor, sensory and autonomic deficits and for which there is currently no adequate treatment. Following the initial trauma to the spinal cord, complex pathological interactions lead to a cascade of secondary damage which results in the establishment of a peri-lesional area which potently inhibits axonal growth and spinal repair (Hagg and Oudega, 2006). This is one of the main reasons for the lack of functional recovery following SCI. Chondroitin sulphate proteoglycans (CSPGs) are known to contribute to the intensely inhibitory environment present at the lesion site, where they are heavily upregulated following SCI, preventing effective regeneration or repair of the damaged spinal cord (Cregg et al., 2014). In addition to being upregulated at the injury site, CSPGs are also present throughout the central nervous system as part of the extracellular matrix. The use of the bacterial enzyme chondroitinase ABC (ChABC) to breakdown CSPGs has repeatedly been shown to have beneficial effects on spinal repair following traumatic injury by numerous different research groups and in a number of different animal species and injury models (Ramer et al., 2014; Garcia-Alias et al., 2009; Bradbury et al., 2002; Alilain et al., 2011).

We have recently demonstrated that using a lentiviral vector to deliver a modified mammalian-compatible ChABC gene to the adult mammalian spinal cord results in endogenous expression of active enzyme, leading to long-term and widespread degradation of CSPGs (Bartus et al., 2014). Moreover, this single delivery gene therapy approach resulted in extensive remodelling of the extracellular matrix and alterations to the post-injury inflammatory response. These changes were associated with dramatic improvements in tissue pathology, conduction of long distance spinal sensory axons and skilled hindlimb locomotion following a thoracic contusion injury, an injury model in which treatment with bacterial ChABC enzyme had previously met with limited success (Iseda et al., 2008; Tom et al., 2009).

We now aim to determine if this treatment can be successfully translated to a more clinically relevant cervical contusion injury model. Since replication is important for the SCI field (Steward et al., 2012), we will assess whether we can replicate our previous findings in a higher level more severe injury model affecting forelimb function. Assessing the efficacy of our treatment in this model is important since just over half of all human SCIs occur at the cervical level, making it the most common injury level (followed by thoracic injury which represents ~ 35% of SCI patients; www.nscisc.uab.edu) and over a quarter of all spinal injured patients have suffered from a contusive-type injury at this level. Therefore, cervical contusion models are highly relevant pre-clinical models of SCI. These models also allow the study of impairments to upper limb function which is one of the highest priority functions for patients with SCI (Anderson, 2004). Our results show that ChABC gene therapy leads to significant anatomical and functional repair following cervical contusion injury. Importantly treatment led to significant improvements in upper limb and hand function, shown using assessments of skilled locomotion and forelimb grip tasks.

## 2 Methods

### 2.1 Animals

Adult male Sprague-Dawley rats (230–250 g; Harlan laboratories) were used (housed under a 12 hour light/dark cycle with free access to food and water). All experimental procedures were performed in accordance with the U.K. Animals (Surgical Procedures) Act 1996. Behavioural testing, electrophysiology and histological analyses were all performed by experimenters blinded to treatment. Randomisation to assign animals to treatment groups was carried out by an individual playing no further role in the study, as were intraspinal injections of each treatment. A total of 24 animals were used with n = 8 assigned to each treatment group (contusion only, contusion + LV-GFP and contusion + LV-ChABC). All animals were assessed behaviourally and n = 5/group were used for the electrophysiological and histological assessments.

### 2.2 Chondroitinase gene and lentiviral vectors

The *Proteus vulgaris* ChABC gene was modified with mutations targeted to remove five cryptic N-glycosylation sites and addition of a mammalian signal sequence and resynthesised with mammalian preferred codons to make a mammalian-compatible engineered ChABC gene (Muir et al., 2010). The modified ChABC cDNA was subcloned into a lentiviral transfer vector (termed LV-ChABC) with the mouse phosphoglycerate kinase promoter, as previously described (Bartus et al., 2014). The resulting vector was integrating, self-inactivating and pseudotyped with VSV-G. Viral particles were concentrated by ultracentrifugation and titered by a p24 antigen ELISA assay. The viral titer was 479 μg/ml of P24, corresponding to ~ 10^6^ TU/μl. A control lentiviral vector (termed LV-GFP) was generated from the same transfer vector containing the cDNA coding for GFP, with a viral titer of 346 μg/ml of P24.

### 2.3 Contusion injury surgery and treatment

Male adult rats were anaesthetized (ketamine, 60 mg/kg, and medetomidine, 0.25 mg/kg), the cervical region of their backs were shaved and cleansed with iodine and core temperature was maintained close to 37 °C using a self-regulating heated blanket. A laminectomy was then performed at vertebral level C5 to expose the underlying spinal cord. Animals were then positioned and stabilised with the impactor probe of an Infinite Horizon impactor (Precision Systems Instrumentation, Lexington, KY) positioned 3–4 mm above the exposed spinal cord. The impactor probe then delivered an impact force of 225 kdyne to the spinal cord, inducing a moderate cervical (C5) contusion injury. Following injury, rats designated to a contusion only group had their wounds sutured and anaesthesia was then reversed using atipamezole hydrochloride (1 mg/kg administered i.p.). All other animals received 0.5 μl intraspinal injections of either LV-GFP or LV-ChABC immediately rostral and caudal to the contusion injury (one injection either side). Injections were delivered at a rate of 200 nl/min using an ultra micropump III (World Precisions Instrumentation, Europe). Following intraspinal injection, wounds were closed and anaesthesia was reversed. All animals received post-operative analgesia (carprofen mg/kg) for two days, saline for rehydration (3–5 ml) for three days, and a one week course of antibiotics (baytril; mg/kg).

### 2.4 Behavioural assessments

Animals were trained in all behavioural tasks for one week prior to surgery and were assessed on the day prior to surgery to provide baseline data for each task. Assessments were then carried out on a weekly basis post-surgery by an experimenter blinded to treatment group.

Skilled locomotion and sensorimotor integration were assessed using the horizontal ladder test. This involved counting the total number of both forelimb and hindlimb footslips made by each animal over the course of three runs across a 1 m ladder with unevenly spaced rungs.

Forelimb grip-strength was assessed using a forelimb grip-strength meter (Linton Instrumentation). This involved rats grasping onto a metal bar with their forepaws, which was attached to a force transducer, and slowly being pulled backwards with gradually increasing force until the rat could no longer hold on and would then release the bar. The grip-strength meter gave a readout of the maximum force applied to the force transducer (i.e. the force at which the rat could no longer grip the bar) and this was recorded. An average was taken from three grip-strength readouts per session.

For the inclined plane task animals were placed horizontally on a rubberised surface (to provide traction) initially held at an angle of 40°. If the rat could hold its position, with no signs of slipping, for 4 s then the incline was increased by 5°. This process was repeated until the incline reached an angle at which the rat could no longer stay stationary and began to slip. This was repeated twice more and an average angle of incline at which the rat began to slip was calculated from the three scores.

### 2.5 Electrophysiological assessment of sensory function

Axonal conduction was assessed by antidromic stimulation of sensory fibres in the dorsal columns in terminal electrophysiology experiments, using a method adapted from a previous study (James et al., 2011). The spinal cord was exposed from C2 to T1 and T13 to L5 (vertebral levels) in urethane-anaesthetized rats (1.25 g/kg). Antidromic unitary activity was recorded and quantified from teased dorsal root filaments (left and right L3–S1 roots), whilst first stimulating the entire dorsal columns 5 mm below and then 5 mm above the injury site. Conduction through the injury site was measured by calculating the number of single units present when stimulating above the injury site as a percentage of the total number of single units when stimulating below. Stimulation was continuous during recording sessions and was carried out using 0.2 ms duration, square wave pulses at a frequency of 1 Hz and an incrementally increasing intensity (0–800 μA). Stimulation was maximal by 400–500 μA (i.e. no further unitary activity could be recruited), but continued to be increased to 800 μA to ensure that no high-threshold fibres were undetected.

### 2.6 Histological assessment of cavity size

Tissue preparation and eriochrome cyanine histochemistry were performed as previously described (James et al., 2011). Sections were examined using a Zeiss Axioskop microscope and pictures were taken of sections at 800 μm intervals throughout the lesion site using a Zeiss AxioCam MRm. Images were analysed using Axiovision software, which allowed the tracing of the spinal cord perimeter and any cavity for each image captured, giving the total area for these measurements in each section. The lesion epicentre was defined as the section from each animal with the largest cavity area and quantification was carried out at 800 μm intervals from 2.4 mm caudal to 2.4 mm rostral to the epicentre. The cavity area was calculated as a percentage of spinal cord area (i.e. area within the tracing of spinal cord perimeter) for each section.

### 2.7 Statistical analysis

All numerical values are reported as mean ± s.e.m. and all statistical analyses were carried out using GraphPad Prism v5 software. Behavioural and histological data were analysed using two-way repeated measures ANOVA, electrophysiological data was analysed by one-way ANOVA. Post-hoc comparisons were carried out where appropriate and all statistical tests are stated in the text.

## 3 Results

### 3.1 Chondroitinase gene therapy promotes recovery of upper limb function

**Fig. 1:**
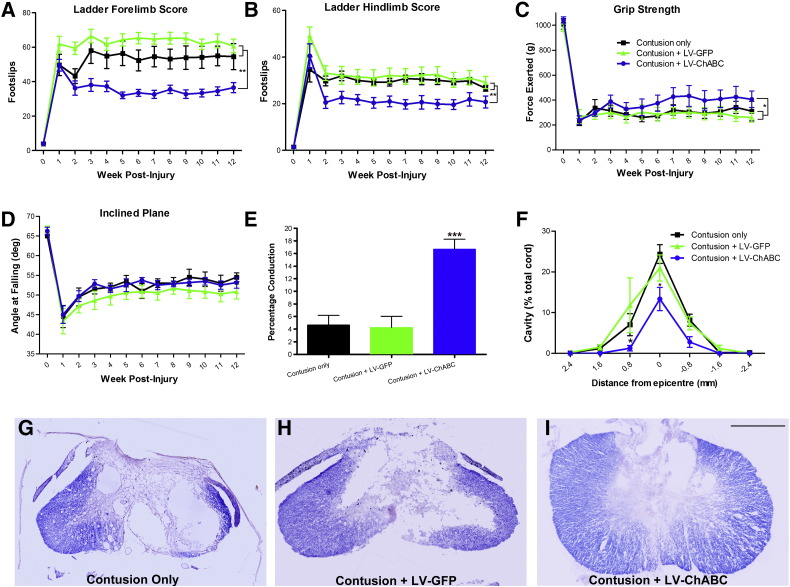
Chondroitinase gene therapy leads to significant improvements in upper limb and hand function, spinal conduction and tissue pathology. LV-ChABC significantly improves recovery of skilled locomotor function in forelimbs (A) and hindlimbs (B) in comparison to contusion only or treatment with LV-GFP (p < 0.01, two-way RM ANOVA, Bonferroni post-hoc). (C) Significant improvements in comparison to both control groups are also detected following assessment of forelimb grip-strength in LV-ChABC treated animals (p < 0.05, two-way RM ANOVA), but no differences are detected in proprioceptive function as assessed by the inclined plane task (D). (E) LV-ChABC treated animals display dramatic improvements in conduction of long distance sensory axons through the cervical contusion injury site when assessed electrophysiologically (p < 0.001, one-way ANOVA, Tukey's post hoc). LV-ChABC treatment (I) results in a significant reduction of cavity size at the injury epicentre and a reduction in its rostral spread (F) when compared with contusion only (G) and LV-GFP treatment (H) (p < 0.05, two-way RM ANOVA, Bonferroni post-hoc). n = 8/group A–D, n = 5/group E and F. Scale bar represents 1 mm (G–I).

Following treatment of a 225 kdyne C5 contusion with LV-ChABC, significant functional improvements in skilled forelimb and hindlimb locomotion were detected when assessed using the horizontal ladder (Fig. 1A and B). Animals treated with LV-ChABC displayed significantly improved functional recovery on the horizontal ladder compared to all other treatment groups at all post-injury time points beyond 3 weeks (p < 0.01; two-way RM ANOVA, Bonferroni post-hoc), such that by 10 weeks post-injury LV-ChABC treated animals were making 33.3 ± 2.2 forelimb and 19.6 ± 2.1 hindlimb slips compared to 54.1 ± 6.1 and 29.4 ± 1.2 for contusion only and 61.7 ± 3 and 30.3 ± 2.8 for contusion + LV-GFP. LV-ChABC treated animals also displayed significant improvements in forelimb grip-strength compared to all other treatment groups (Fig. 1C; p < 0.05, two-way RM ANOVA). At 10 weeks post-injury LV-ChABC treated animals were capable of exerting a grip-strength of 409.8 ± 70.3 g, whilst LV-GFP treated and contusion only animals achieved forces of only 296.2 ± 53.1 g and 299.7 ± 42.7 g, respectively. There were no detectable differences between groups when comparing proprioceptive function using the inclined plane task (Fig. 1D). These results indicate that LV-ChABC leads to significant improvements in both upper and lower limb functions following a cervical contusion injury.

### 3.2 Chondroitinase gene therapy leads to improved long distance axonal conduction through a cervical contusion site

Electrophysiological experiments were next performed in order to assess the extent of long distance, axonal conduction across the cervical contusion site. The antidromic activation of sensory dorsal column fibres caudal to the injury site allowed the recording of unitary activity from thin filaments teased from lumbar dorsal roots (James et al., 2011). Conduction through the injury site could then be assessed by switching stimulation to a site just rostral to the lesion and determining the percentage of single units still present in each dorsal root filament. LV-ChABC treated animals displayed significantly improved levels of conduction through the injury site in comparison to all other treatment groups (16.7 ± 1.6% compared to 4.7 ± 1.6% and 4.3 ± 1.8% for contusion only and contusion + LV-GFP, respectively; p = 0.001, one-way ANOVA, Tukey's post-hoc; Fig. 1E). These findings highlight a dramatic improvement in the functionality of the spinal cord's major sensory pathway following cervical contusion and treatment with LV-ChABC.

### 3.3 Chondroitinase gene therapy leads to reduced tissue pathology

In order to assess anatomical changes due to treatment with LV-ChABC, erichrome cyanine staining was performed on histological tissue sections to allow quantification of cavity formation at the lesion epicentre and the rostro-caudal spread of tissue damage (Fig. 1F–I). Overall cavity development in LV-ChABC treated animals (Fig. 1I) was significantly reduced in comparison with contusion only (Fig. 1G) and LV-GFP (Fig. 1H) treated animals (p < 0.01 two-way RM ANOVA; Fig. 1F). The most significant reduction in cavity size was apparent at the lesion epicentre, such that at its largest the cavity in LV-ChABC treated animals was approximately half the size of the cavities which developed in the two control groups (13.3 ± 2.9% compared to 24.4 ± 2.3% and 21.3 ± 3.3% for contusion only and LV-GFP, respectively; p < 0.05, Bonferroni post-hoc; Fig. 1F). In addition, there was a reduction in the spread of cavity development from the lesion epicentre in LV-ChABC treated animals such that there was minimal cavity present at 0.8 mm rostral or caudal to the epicentre in LV-ChABC treated animals, whilst in contusion only and LV-GFP treated animals cavities were still approximately 10% of the total cord area in these tissue sections (Fig. 1F; p < 0.05 at 0.8 mm rostral of epicentre, Bonferroni post-hoc).

## 4 Discussion

Here we demonstrate that ChABC gene therapy is effective when used in a model of cervical contusion injury. This marks an important step in demonstrating the potential for this treatment to be further developed towards clinical translation. Behavioural analyses demonstrated significant improvements in both forelimb and hindlimb function during skilled locomotion as well as improved forelimb grip-strength. Additionally, terminal electrophysiological experiments highlighted dramatically improved functionality in the main sensory pathway of the spinal cord. These functional improvements were complimented by anatomical observations indicating that there was significantly reduced cavity formation in animals receiving ChABC gene delivery. That this treatment has now been shown to lead to functional and pathological improvements in contusion injuries at thoracic (Bartus et al., 2014) or cervical levels indicates that the effects of LV-ChABC are robust, a positive sign if this treatment is to be successfully translated. Furthermore, the use of a contusion injury model at cervical level in this study is of critical importance, as this is the most frequently occurring SCI in humans (Jackson et al., 2004) (www.nscisc.uab.edu).

The observation of improved grip-strength in LV-ChABC treated animals is significant when considering the potential translatability of this treatment, since cervical SCI patients list improved upper limb and hand functions as one of their highest priorities (Anderson, 2004). These functions are both reflected well in the grip-strength test, with improved paw function required to grip the apparatus properly and improved upper limb function required to reach increased forces of grip-strength. Whilst significant functional improvements were detected using the horizontal ladder and grip-strength tasks, no such differences were observed using the inclined plane task. Whilst this may simply be a reflection of a lack of sensitivity using the inclined plane task, it may well indicate that spinal pathways important for each of these tasks are affected to differing degrees. For instance, electrophysiological experiments showed that there were dramatic improvements in the functionality of the ascending sensory pathway; a pathway which, along with the corticospinal tract, is known to be heavily involved in skilled locomotor function as well as forelimb grip-strength (Sedy et al., 2008). Conversely, neither of these pathways are thought to be important in performance on the inclined plane task (Sedy et al., 2008). It would therefore be interesting to assess if any improvements in pathways important in inclined plane performance (such as vestibulospinal and reticulospinal tracts) could be detected electrophysiologically in order to determine if LV-ChABC is differentially affecting various spinal pathways.

Thus, we present novel data that chondroitinase gene therapy leads to significant improvements in important functional and anatomical outcome measures using a clinically representative cervical contusion injury model. These results have important implications for the further development of this treatment, indicating that if current attempts to improve its safety profile are successful, assessment in a larger animal model (such as porcine, canine or primate) is likely to be an important next step towards clinical translation (Ramer et al., 2014). Another interesting future direction for this work will be to combine chondroitinase gene therapy with a regime of rehabilitative techniques aimed at further enhancing the observed improvements to upper limb and hand function. This could be achieved through repetitive electrical activation of spinal pathways important to upper limb function in rodents (e.g. corticospinal tract) via implanted electrodes (Carmel et al., 2010), in addition to more traditional behavioural rehabilitative techniques specifically targeting and reinforcing complex movements of the upper limbs (such as skilled reaching tasks) (Garcia-Alias et al., 2009). Efficacy of chondroitinase gene therapy treatment has now been demonstrated in clinically relevant contusion injury models at both thoracic and cervical levels. It will now be important to determine if similar effects are observed in larger animal models and if its efficacy can be further enhanced by combination with additional therapeutic strategies. These findings show promise for the further development of chondroitinase gene therapy, as well as other strategies which target CSPGs (Lang et al., 2015), as potential therapeutics for restoring function following spinal cord injury.
